# *TERT* promoter mutation confers favorable prognosis regardless of 1p/19q status in adult diffuse gliomas with *IDH1*/*2* mutations

**DOI:** 10.1186/s40478-020-01078-2

**Published:** 2020-11-23

**Authors:** Hideyuki Arita, Yuko Matsushita, Ryunosuke Machida, Kai Yamasaki, Nobuhiro Hata, Makoto Ohno, Shigeru Yamaguchi, Takashi Sasayama, Shota Tanaka, Fumi Higuchi, Toshihiko Iuchi, Kuniaki Saito, Masayuki Kanamori, Ken-ichiro Matsuda, Yohei Miyake, Kaoru Tamura, Sho Tamai, Taishi Nakamura, Takehiro Uda, Yoshiko Okita, Junya Fukai, Daisuke Sakamoto, Yasuhiko Hattori, Eriel Sandika Pareira, Ryusuke Hatae, Yukitomo Ishi, Yasuji Miyakita, Kazuhiro Tanaka, Shunsaku Takayanagi, Ryohei Otani, Tsukasa Sakaida, Keiichi Kobayashi, Ryuta Saito, Kazuhiko Kurozumi, Tomoko Shofuda, Masahiro Nonaka, Hiroyoshi Suzuki, Makoto Shibuya, Takashi Komori, Hikaru Sasaki, Masahiro Mizoguchi, Haruhiko Kishima, Mitsutoshi Nakada, Yukihiko Sonoda, Teiji Tominaga, Motoo Nagane, Ryo Nishikawa, Yonehiro Kanemura, Aya Kuchiba, Yoshitaka Narita, Koichi Ichimura

**Affiliations:** 1 Division of Brain Tumor Translational Research, National Cancer Center Research Institute, 5-1-1, Tsukiji, Chuo-ku, Tokyo 104-0045 Japan; 2 Department of Neurosurgery, Osaka University Graduate School of Medicine, 2-2, Yamadaoka, Suita-City, Osaka 565-0871 Japan; 3 Department of Neurosurgery and Neuro-Oncology, National Cancer Center Hospital, 5-1-1, Tsukiji, Chuo-ku, Tokyo 104-0045 Japan; 4 Biostatistics Division, Center for Research Administration and Support, National Cancer Center, 5-1-1, Tsukiji, Chuo-ku, Tokyo 104-0045 Japan; 5 Department of Pediatric Hematology and Oncology, Osaka City General Hospital, 2-13-22, Miyakojima-hondori, Miyakojima-ku, Osaka-City, Osaka 534-0021 Japan; 6 Department of Neurosurgery, Graduate School of Medical Sciences, Kyushu University, 3-1-1 Maidashi, Higashi-ku, Fukuoka-City, Fukuoka 812-8582 Japan; 7 Department of Neurosurgery, Faculty of Medicine, Hokkaido University, North 15 West 7, Kita-ku, Sapporo-City, Hokkaido 060-8638 Japan; 8 Department of Neurosurgery, Kobe University Graduate School of Medicine, 7-5-2, Kusunoki-cho, Chuo-ku, Kobe-City, Hyogo 650-0017 Japan; 9 Department of Neurosurgery, Faculty of Medicine, The University of Tokyo, 7-3-1, Hongo, Bunkyo-ku, Tokyo 113-8655 Japan; 10 Department of Neurosurgery, Dokkyo Medical University, 880, Kitakobayashi, Mibu-City, Tochigi 321-0293 Japan; 11 Division of Neurological Surgery, Chiba Cancer Center, 666-2 Nitonacho, Chuo-ku, Chiba-City, Chiba 260-8717 Japan; 12 Department of Neurosurgery, Kyorin University Faculty of Medicine, 6-20-2, Shinkawa, Mitaka-City, Tokyo 181-8611 Japan; 13 Department of Neurosurgery, Tohoku University Graduate School of Medicine, 1-1 Seiryo-machi, Aoba-ku, Sendai-City, Miyagi 980-8574 Japan; 14 Department of Neurosurgery, Faculty of Medicine, Yamagata University, 2-2, Iida-Nishi, Yamagata-City, Yamagata 990-9585 Japan; 15 Department of Neuro-Oncology/Neurosurgery, Saitama Medical University International Medical Center, 1397-1, Yamane, Hidaka-City, Saitama 350-1298 Japan; 16 Department of Neurosurgery, Graduate School of Medicine, Yokohama City University, 3-9, Fukuura, Kanazawa-ku, Yokohama-City, Kanagawa 236-0004 Japan; 17 Department of Neurosurgery, Tokyo Medical and Dental University, 1-5-45, Yushima, Bunkyo-ku, Tokyo 113-8519 Japan; 18 Department of Neurosurgery, Graduate School of Medical Science, Kanazawa University, 13-1, Takara-machi, Kanazawa-City, Ishikawa 920-8641 Japan; 19 Department of Neurosurgery, Osaka City University Graduate School of Medicine, 1-5-7, Asahi-machi, Abeno-ku, Osaka-City, Osaka 545-8586 Japan; 20 Department of Neurosurgery, National Hospital Organization Osaka National Hospital, 2-1-14 Hoenzaka, Chuo-ku, Osaka-City, Osaka 540-0006 Japan; 21 Department of Neurosurgery, Osaka International Cancer Institute, 3-1-69, Otemae, Chuo-ku, Osaka-City, Osaka 541-8567 Japan; 22 Department of Neurological Surgery, Wakayama Medical University, 811-1, Kimiidera, Wakayama-City, Wakayama 641-0012 Japan; 23 Department of Neurosurgery, Hyogo College of Medicine, 1-1 Mukogawa, Nishinomiya-City, Hyogo 663-8501 Japan; 24 Department of Neurological Surgery, Okayama University Graduate School of Medicine, Dentistry and Pharmaceutical Sciences, 2-5-1 Shikata-cho, Kita-ku, Okayama-City, Okayama 700-8558 Japan; 25 Department of Neurosurgery, Keio University School of Medicine, 35, Shinano-machi, Tokyo, Shinjuku-ku 160-8582 Japan; 26 Department of Neurosurgery, Tokyo Metropolitan Komagome Hospital, 3-18-22, Honkomagome, Bunkyo-ku, Tokyo 113-8677 Japan; 27 Department of Biomedical Research and Innovation Research, Institute for Clinical Research, National Hospital Organization Osaka National Hospital, 2-1-14, Hoenzaka, Chuo-ku, Osaka-City, Osaka 540-0006 Japan; 28 Department of Neurosurgery, Kansai Medical University, 3-1, Shinmachi 2 Chome, Hirakata-City, Osaka 573-1191 Japan; 29 Department of Pathology and Laboratory Medicine, National Hospital Organization, Sendai Medical Center, 2-11-12, Miyagino, Miyagino-ku, Sendai-City, Miyagi 983-8520 Japan; 30 Central Clinical Laboratory, Hachioji Medical Center, Tokyo Medical University, 1163, Tatemachi, Hachioji-City, Tokyo 193-0998 Japan; 31 Department of Laboratory Medicine and Pathology (Neuropathology), Tokyo Metropolitan Neurological Hospital, 2-6-1 Musashidai, Fuchu, Tokyo 183-0042 Japan

**Keywords:** *IDH1/2*, *TERT*, 1p/19q codeletion, *CDKN2A*, Glioma

## Abstract

*TERT* promoter mutations are commonly associated with 1p/19q codeletion in IDH-mutated gliomas. However, whether these mutations have an impact on patient survival independent of 1p/19q codeletion is unknown. In this study, we investigated the impact of *TERT* promoter mutations on survival in IDH-mutated glioma cases. Detailed clinical information and molecular status data were collected for a cohort of 560 adult patients with IDH-mutated gliomas. Among these patients, 279 had both *TERT* promoter mutation and 1p/19q codeletion, while 30 had either *TERT* promoter mutation (n = 24) or 1p/19q codeletion (n = 6) alone. A univariable Cox proportional hazard analysis for survival using clinical and genetic factors indicated that a Karnofsky performance status score (KPS) of 90 or 100, WHO grade II or III, *TERT* promoter mutation, 1p/19q codeletion, radiation therapy, and extent of resection (90–100%) were associated with favorable prognosis (*p* < 0.05). A multivariable Cox regression model revealed that *TERT* promoter mutation had a significantly favorable prognostic impact (hazard ratio = 0.421, *p* = 0.049), while 1p/19q codeletion did not have a significant impact (hazard ratio = 0.648, *p* = 0.349). Analyses incorporating patient clinical and genetic information were further conducted to identify subgroups showing the favorable prognostic impact of *TERT* promoter mutation. Among the grade II-III glioma patients with a KPS score of 90 or 100, those with *IDH*-*TERT* co-mutation and intact 1p/19q (n = 17) showed significantly longer survival than those with *IDH* mutation, wild-type *TERT*, and intact 1p/19q (n = 185) (5-year overall survival, 94% and 77%, respectively; *p* = 0.032). Our results demonstrate that *TERT* promoter mutation predicts favorable prognosis independent of 1p/19q codeletion in IDH-mutated gliomas. Combined with its adverse effect on survival among IDH-wild glioma cases, the bivalent prognostic impact of *TERT* promoter mutation may help further refine the molecular diagnosis and prognostication of diffuse gliomas.

**Electronic supplementary material:**

The online version of this article (10.1186/s40478-020-01078-2) contains supplementary material, which is available to authorized users.

## Introduction

Recent advances in molecular genetics over the last decade have facilitated the integration of molecular markers into the diagnosis of brain tumors. The revised 4th edition of the World Health Organization (WHO) classification of Tumours of the Central Nervous System (the CNS WHO 2016) incorporated molecular diagnosis in the diagnostic criteria for the first time in its history [17]. The *IDH1/2* (*IDH*) status plays a crucial role in defining adult diffuse gliomas in the current diagnostic system. *IDH* mutation and 1p/19q codeletion are necessary and sufficient to make the diagnosis of oligodendrogliomas regardless of the histology. The 1p/19q codeletion is the key diagnostic marker to delineate oligodendrogliomas and distinguish them from astrocytomas in IDH-mutated tumors. Although the consortium to inform molecular and practical approaches to CNS tumor taxonomy-not official WHO (cIMPACT-NOW) recommended a practical diagnostic scheme for diffuse gliomas based on the results of ATRX/p53 immunohistochemistry [16], the *ATRX* status is only a surrogate and sometimes inconclusive [24].

*TERT* promoter mutations are common in oligodendrogliomas and glioblastomas [4]. We and others have shown that *TERT* promoter mutations are frequently observed (> 90%) in oligodendrogliomas with mutant *IDH* and 1p/19q codeletion, and that the presence of *TERT* promoter mutations is associated with favorable outcomes in IDH-mutated gliomas [6, 14, 15]. These findings strongly suggest that *TERT* promoter mutations may serve as an alternative diagnostic marker for oligodendrogliomas when combined with the *IDH* status. Another aspect of *TERT* promoter mutation is that this alteration without accompanying *IDH* mutation suggests clinically and biologically aggressive characteristics comparable with those of glioblastomas when found in histologically diagnosed as diffuse gliomas [6]. The presence of the *TERT* promoter mutation indicates the underestimation of the tumor grades when observed in grade II–III diffuse gliomas without *IDH* mutation. cIMPACT-NOW Update 3 recommended *TERT* promoter mutations as one of the three criteria (the other two being either *EGFR* amplification or combined whole chromosome 7 gain/chromosome 10 loss) to diagnosis “Diffuse astrocytic glioma, IDH-wildtype, with molecular features of glioblastoma, WHO grade IV” [8]. Thus, *TERT* promoter mutations serve as a diagnostic marker to delineate histologically verified IDH-wild diffuse astrocytomas with poor outcome comparable with glioblastomas. Evaluation of this marker is becoming an essential part of the routine diagnosis for diffuse astrocytic tumors with wildtype *IDH*. The bivalent impact of *TERT* promoter mutations on glioma biology depends on the *IDH* status, as such, we have previously proposed a molecular classification based on the *IDH* and *TERT* status, which can efficiently identify diffuse astrocytomas and oligodendrogliomas [6].

In this study, in order to further understand the diagnostic and prognostic value of *TERT* promoter mutation, we examined the impact of *TERT* promoter mutations on survival in a series of IDH-mutated glioma cases using a large retrospective tumor cohort. Our results showed that *TERT* promoter mutations predict favorable prognosis regardless of 1p/19q status in IDH-mutated gliomas. We propose that *TERT* promoter mutations are bivalent diagnostic and prognostic markers for adult diffuse gliomas.

## Materials and methods

### Patient cohorts

Two cohorts were integrated for this retrospective study: one that was analyzed in our previous study [6] and the other was newly collected for this study. The inclusion criteria for both cohorts were as follows: histological diagnosis of IDH1/2-mutated diffuse glioma, 18 years of age or older, clinical data obtained for survival analysis, and availability of genomic DNA extracted from frozen tissues at the time of initial surgery before chemoradiation. Out of the 951 cases analyzed in the previous study, 286 cases with *IDH* mutations from 13 institutions were enrolled in this study, and their clinical data were updated. The new cohort included 274 cases from 8 institutions. Thus, in total, 560 cases of IDH-mutated diffuse glioma were analyzed in the present study.

### Clinical data and histology

Detailed clinical information including patient age, preoperative Karnofsky Performance status (KPS) score, tumor location, extent of resection (EOR), and adjuvant therapy following the initial surgery was obtained from patient medical records. Local histological diagnosis made at each institution was obtained. The majority of tumors (540/560 cases, 96%) were operated on before May 2016; thus, the histopathological diagnosis was almost entirely made according to the CNS WHO 2007 in each center. In this study, an integrated diagnosis was determined by incorporating molecular data and histological diagnosis, which made the diagnosis compatible with the CNS WHO 2016. WHO grade IV tumors with *IDH* mutation and 1p/19q codeletion were reclassified as grade III based on the current diagnostic criteria which classifies these as anaplastic oligodendrogliomas with *IDH* mutation and 1p/19q codeletion. The histological diagnosis of the original data is also provided in Additional file 1: Table S1 to show the relationship between molecular features and microscopic findings. For survival analysis, patients were subdivided into two groups based on age (≤ 50 or > 50 years) and preoperative KPS score (< 90 or ≥ 90%). These cutoffs were based on the University of California at San Francisco Low-Grade Glioma Prognostic Scoring System, established and validated by a multi-institutional outcome analysis of cohorts consisting of low-grade gliomas [9, 10]. The EOR was based on the report made by the surgeons in the operation record of the initial surgery.

### Molecular analysis

Genomic DNA from frozen tumor tissues was extracted using the DNeasy Blood & Tissue Kit (Qiagen, Tokyo, Japan), according to the manufacturer’s protocol. Molecular testing was performed as previously described. Briefly, the mutational status of *IDH1/2* and *TERT* promoter was tested by Sanger sequencing and/or pyrosequencing [5, 6]. The 1p/19q status was examined by a multiplex ligation-dependent probe amplification (MLPA) [6], microsatellite analysis [23, 27], or microarray-based comparative genomic hybridization [1, 4]. The results of fluorescence in situ hybridization were not included to avoid ambiguity of judgment that could be caused by partial deletions in 1p and/or 19q [13]. The copy number of the *CDKN2A* locus was also determined by MLPA [6].

### Statistical analysis

Categorized data were compared between molecular groups using a Chi square test. Survival was estimated by the Kaplan–Meier method and compared using a log-rank test. Hazard ratios (HRs) and 95% confidence intervals (CIs) were estimated using the Cox regression model in patients with complete clinical information (n = 557). Overall survival (OS) was defined as the duration from the date of initial surgery to that of either death or the last follow-up, with a censoring cutoff date of 30 September 2017. Patients alive at the last follow-up were considered censored during the survival analysis. Differences were considered significant if the *p* value was < 0.05. All statistical analyses were performed using JMP Pro version 14 software (SAS Institute, Cary, NC, USA).

## Results

### Patient characteristics

A total of 560 diffuse glioma patients with confirmed *IDH* mutations were analyzed in the present study. The mean age of all patients was 43.5 years (range 18–82 years). Most patients were diagnosed with lower grade gliomas (527 cases, 94.1%) based on CNS WHO 2016. Approximately 82% (460 cases) of patients had only minor symptoms or no complaints (KPS score 90 or 100). The median follow-up period was 64.7 months (range; 0.85 to 208 months). *TERT* promoter mutation and 1p/19q codeletion were found in 303 (54.1%) and 285 (50.9%) cases, respectively. Among them, 279 cases harbored both *TERT* mutation and 1p/19q codeletion, while 30 cases had either *TERT* mutation (n = 24) or 1p/19q codeletion (n = 6). The remaining 251 IDH-mutant cases had neither of them. Infratentorial tumors with *IDH* mutation were extremely rare (n = 3) and harbored neither of *TERT* promoter mutation nor 1p/19q codeletion. The patients’ clinical background and molecular status are summarized in Table 1, and detailed information for each case is provided in Additional file 1: Table S1.

**Table 1:** Patient characteristics (n = 560)

*IDH*	All	mut	mut	mut	mut
*TERT*		mut	mut	wt	wt
1p/19q		codel	intact	codel	intact
Total (n)	560	279	24	6	251
Mean age (y.o.)	43.5	46.4	41.8	48.2	40.3
− 50	399	178	18	3	200
> 50	161	101	6	3	51
M/F	317/243	162/117	14/10	5/1	136/115
WHO grade^a^					
II	287	145	13	4	125
III	240	134	6	2	98
IV	33	0	5	0	28
Integrated diagnosis^a^					
DA	138	0	13	0	125
AA	104	0	6	0	98
OL	149	145	0	4	0
AO	136	134	0	2	0
GBM	33	0	5	0	28
KPS					
90–100	460	238	20	6	196
< 90	98	41	4	0	53
nd	2	0	0	0	2
Location					
Supratentorial	557	279	24	6	248
Infratentorial	3	0	0	0	3
*CDKN2A*					
Homo Del	19	3	1	1	14
Non-Del	365	187	17	3	158
nd	176	89	6	2	79
RT					
(+)	318	137	17	3	161
(−)	241	141	7	3	90
nd	1	1	0	0	0
Chemo					
(+)	379	210	16	3	150
(−)	180	68	8	3	101
nd	1	1	0	0	0
EOR					
90–100%	329	179	15	2	133
< 90%	231	100	9	4	118

AA, anaplastic astrocytoma, IDH-mutant; AO, anaplastic oligodendroglioma, IDH-mutant and 1p/19q-codeleted; Chemo, Chemotherapy; codel, codeleted; DA, diffuse astrocytoma, IDH-mutant; Del, Deletion; EOR, extent of resection; F, female; GBM, glioblastoma, IDH-mutant; Homo, Homozygous; nd, no data; KPS, Karnofsky Performance Status; M, male; mut, mutated; OL, oligodendrolioma, IDH-mutant and 1p/19q-codeleted; RT, radiation therapy; y.o., years old; wt, wild-type

^a^Diagnosis based on CNS WHO2016

### *TERT* promoter mutation has a favorable impact on survival, independent of 1p/19q status

The results of a univariable Cox proportional hazard analysis for survival using each clinical and genetic factor are shown in Table 2. KPS score, WHO grade, *TERT* promoter status, 1p/19q status, adjuvant radiation therapy, and EOR were significantly associated with survival. The Kaplan–Meier survival curves also showed that both *TERT* promoter mutation and 1p/19q codeletion were strongly associated with a favorable prognosis in IDH-mutated gliomas (Additional file 2: Fig. S1A and B).

**Table 2:** Univariable and multivariable Cox regression analysis for survival (n = 557)

	n	Univariable			Multivariable		
		HR	95% C.I.	*p* value	HR	95% C.I.	*p* value
Sex							
M	315	0.832	0.592–1.169	0.289	1.076	0.759–1.527	0.680
F	242	Ref			Ref		
Age							
− 50	397	Ref			Ref		
> 50	160	1.361	0.945–1.960	0.098	1.555	1.051–2.300	0.027
KPS							
90–100	459	Ref			Ref		
< 90	98	2.833	1.971–4.074	< 0.0001	1.706	1.136–2.562	0.010
WHO grade^a^							
II	285	Ref			Ref		
III	239	1.167	0.808–1.687	0.410	1.198	0.778–1.845	0.413
IV	33	8.646	5.135–14.558	< 0.0001	5.761	2.978–11.145	< 0.0001
*TERT*							
wt	255	Ref			Ref		
mut	302	0.278	0.191–0.404	< 0.0001	0.421	0.178–0.998	0.0494
1p/19q							
Non-codel	273	Ref			Ref		
Codel	284	0.286	0.195–0.419	< 0.0001	0.648	0.262–1.604	0.349
RT							
(−)	240	Ref			Ref		
(+)	317	1.555	1.084–2.232	0.017	0.847	0.531–1.349	0.484
Chemo							
(−)	179	Ref			Ref		
(+)	378	1.252	0.857–1.829	0.246	1.299	0.769–2.196	0.329
EOR							
90–100%	326	0.487	0.346–0.685	< 0.0001	0.513	0.359–0.735	0.0003
< 90%	231	Ref			Ref		

Chemo, Chemotherapy; C.I., Coefficient interval; codel, codeleted; EOR, extent of resection; F, female; HR, hazard ratio; KPS, Karnofsky Performance Status; M, male; mut, mutant; Ref, Reference; RT, radiation therapy; wt, wild-type

^a^Diagnosis based on CNS WHO2016

We further conducted a multivariable analysis using the Cox regression model for survival incorporating clinical factors and treatments (Table 2). *TERT* promoter mutation had a survival benefit with an HR of 0.421 (95% CI: 0.178–0.998, *p* = 0.0494), whereas the impact of 1p/19q status was not apparent (HR 0.648; 95% CI 0.262–1.604; *p* = 0.349). To elucidate the subgroup with a benefit or disadvantage from the *TERT* promoter mutation, we evaluated the HR of *TERT* promoter mutation by subgroup analysis in 1p/19q codeleted and intact groups, respectively. For this subgroup analysis, we performed multivariable analysis of the clinical factors that were considered to be significant in the initial multivariable analysis of all cases. The point estimates of HR consistently indicated the favorable impact of *TERT* promoter mutation regardless of the combination of clinical factors in both the 1p/19q codeleted and intact groups (Additional file 1: Table S2A-L and S3A-L).

### The prognosis of the *IDH*-*TERT* co-mutated-1p/19q intact group was comparable to that of the *IDH*-*TERT* co-mutated-1p/19q codeleted group among WHO grade II-III cases

For the purpose of investigating the impact of *TERT* promoter mutation and 1p/19q codeletion on survival in IDH-mutated glioma cases, the patient cohort was divided into four groups dictated by *TERT* and 1p/19q statuses. The patient details of each group are shown in Table 1. The original histological diagnoses of the *TERT*-mutated-1p/19q intact (IDH-mutated) group included various histological types and contained fewer pure oligodendroglial tumors (9 out of 24 cases), while most *TERT*-wildtype-1p/19q codeleted tumors were histologically diagnosed as pure oligodendroglial tumors (5 out of 6 cases) (Additional file 1: Table S1).

**Fig. 1:**
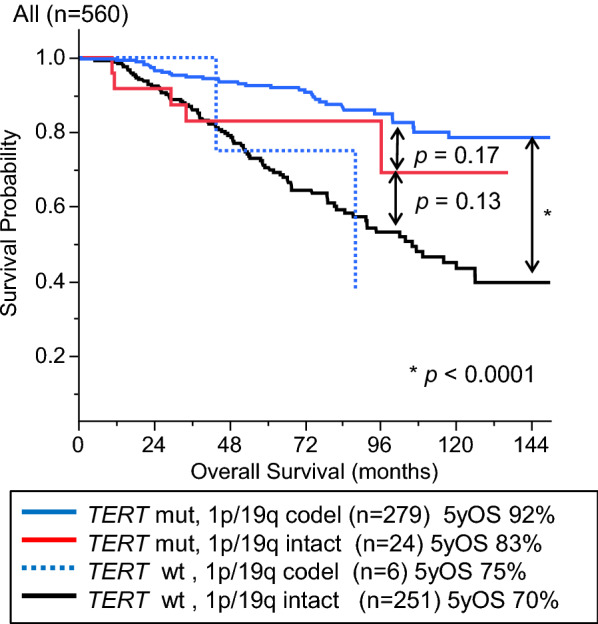
Kaplan-Meier analysis for OS in IDH-mutated gliomas when stratified by *TERT* and 1p/19q status (n = 560). *TERT*-mutated-1p/19q intact group showed an intermediate survival curve between *TERT*-mutated-1p/19q codeleted and *TERT*-wildtype-1p/19q intact groups, although the differences were not statistically significant. codel, codeleted; OS, overall survival; and 5yOS, 5-year overall survival

The *TERT*-mutated-1p/19q intact group, including all histological grades, showed intermediate survival between that of the *TERT*-mutated-1p/19q codeleted and *TERT*-wildtype-1p/19q intact groups; however, the differences were not statistically significant (*p* = 0.17 and 0.13, respectively) (Fig. 1).

**Fig. 2:**
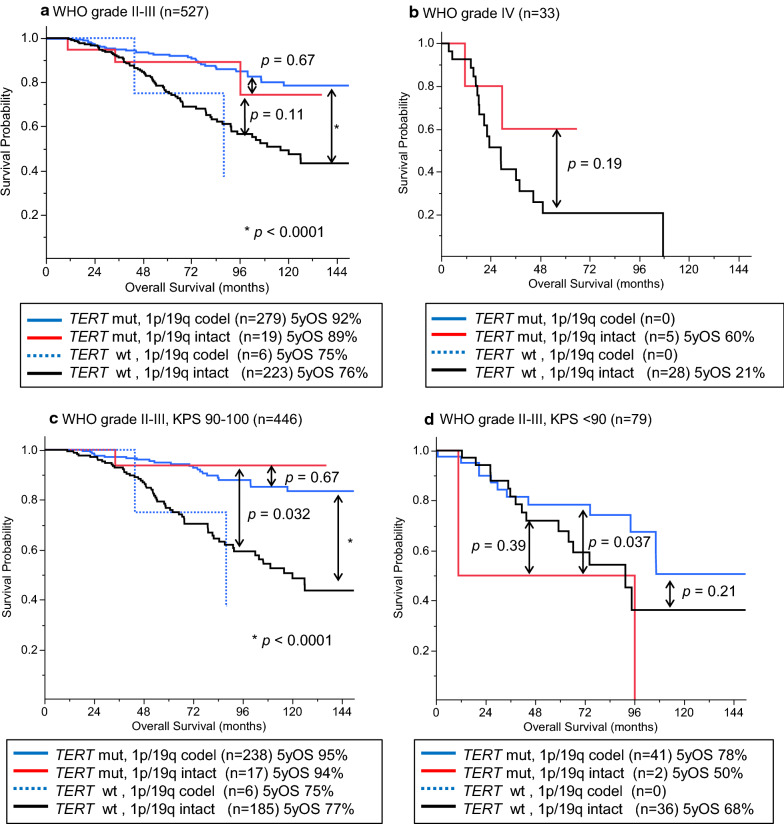
Survival impact of *TERT* and 1p/19q statuses in grade II–III gliomas when stratified by Karnofsky Performance Status (KPS) scores. **a** OS of WHO grade II–III cases (n = 527) stratified by the *TERT* and 1p/19q statuses. Survival curves of *TERT*-mutated-1p/19q intact group and *TERT*-mutated-1p/19q codeleted group were mostly overlapping. **b** OS of WHO grade IV cases (n = 33) stratified by the *TERT* and 1p/19q statuses. *TERT*-mutated-1p/19q intact group showed a tendency towards longer survival compared with that of the *TERT*-wildtype-1p/19q intact group although the difference was not significant. **c** *TERT*-mutated-1p/19q intact cases with a high KPS score (90 or 100) had a favorable prognosis comparable to that of the *TERT*-mutated-1p/19q codeleted cases. D. When analyzed in the population with a KPS score below 90, *TERT* mutation without 1p/19q codeletion did not show survival benefit. codel, codeleted; KPS, Karnofsky Performance Status; OS, overall survival.; 5yOS, 5-year overall survival

Further subgroup analysis was performed in the groups of grades II–III and IV because the Kaplan–Meier and Cox proportional hazard analyses demonstrated apparent differences between these grade groups (Additional file 2: Fig. S2 and Table 2). In the grade II-III glioma group, there was a significant difference in survival between the *TERT*-mutated-1p/19q codeleted group and *TERT*-wildtype-1p/19q intact group (*p* < 0.0001) (Fig. 2a). The survival curve of the *TERT*-mutated-1p/19q intact group was close to that of the *TERT*-mutated-1p/19q codeleted group. The *TERT*-mutated-1p/19q intact group showed a tendency towards longer survival than that of the *TERT*-wildtype-1p/19q intact group, although the difference was not statistically significant (*p* = 0.11); this is probably because of the limited number of these rare cases in the cohort (Fig. 2a). The survival curve of the *TERT*-wildtype-1p/19q codeleted group was close to that of the *TERT*-wildtype-1p/19q intact group (Fig. 2a). In the grade IV tumor group, the *TERT*-mutated-1p/19q intact group showed a tendency towards longer survival compared with that of the *TERT*-wildtype-1p/19q intact group, although the difference was not statistically significant (*p* = 0.19) (Fig. 2b). Thus, *TERT* promoter mutations without 1p/19q codeletion seemed to have a favorable impact on survival.

### The favorable prognosis associated with *TERT* promoter mutations independent of 1p/19q codeletion was seen in grade II–III IDH-mutated cases with higher KPS scores (≥ 90)

We further analyzed the effect of *TERT* promoter mutation in IDH-mutated grade II-III tumors with respect to prognosis after stratification by KPS scores. KPS score was a significant prognostic factor among pretreatment parameters (sex, age, and KPS score) in the univariable and multivariable Cox proportional hazard analyses (Table 2). Moreover, when the grade II-III group was subdivided by KPS scores, cases with a good performance status (KPS score of 90-100) showed favorable prognosis compared to those with a KPS score under 90 (*p* = 0.0002) (Additional file 2 Fig. S3). When comparing molecular subgroups, the *TERT*-mutated groups with patients with grade II–III tumors and higher KPS scores (90–100) showed longer survival regardless of 1p/19q status (Fig. 2c). The *TERT*-mutated-1p/19q intact group showed significantly longer survival than that of the group with neither *TERT* promoter mutation nor 1p/19q codeletion (*p* = 0.032), and the survival of the former was comparable with that of the *TERT*-mutated-1p/19q codeleted group (Fig. 2c). The survival curve of the cases with higher KPS scores in the *TERT*-wildtype-1p/19q codeleted group was close to that of the *TERT*-wildtype-1p/19q intact group (Fig. 2c). On the other hand, neither 1p/19q codeletion nor *TERT* promoter mutation was associated with a favorable prognosis in subgroups with a lower KPS score (< 90) (Fig. 2d). Only two *TERT*-mutated-1p/19q intact cases were included in the analysis for low KPS score.

### Histological grade was associated with survival in 1p/19q intact cases but not in 1p/19q codeleted cases

We investigated the difference in the prognostic impact of histological grade on survival between 1p/19q intact and codeleted cases. When subdivided by 1p/19q status, histological grade was not associated with survival in the 1p/19q codeleted cases but was significantly associated with prognosis in the 1p/19q intact IDH-mutated cases (Additional file 2: Fig. S4 A and B).

### *CDKN2A* homozygous deletion was associated with shorter survival and higher histological grade among IDH-mutated-1p/19q intact tumors

We also analyzed the prognostic relevance of *CDKN2A* in IDH-mutated tumor cases. *CDKN2A* status was available for 385 patients. *CDKN2A* homozygous deletion was observed in all molecular groups; however, the majority of deletions were found in those with *TERT*-wildtype-1p/19q intact tumors (Table 1). This alteration was associated with a higher grade (*p* < 0.0001) and a lower KPS score (*p* < 0.0001) compared to those of cases without this alteration. Tumors with *CDKN2A* homozygous deletion and 1p/19q codeletion were rare (4 cases), and as such, their effect on prognosis could not be evaluated (Additional file 2: Fig. S5A). In 1p/19q intact tumors, cases with *CDKN2A* homozygous deletion (n = 15) showed significantly shorter survival than those without this copy number alteration (n = 175) (*p* < 0.0001) (Additional file 2 Fig. S5B). Most of the *CDKN2A* deleted tumors without 1p/19q codeletion showed a higher histological grade (grade II, one case; grade III, five cases; and grade IV, nine cases). When confined to the cases for which *CDKN2A* status was available, an unfavorable prognosis for WHO grade IV cases was retained even after excluding cases with *CDKN2A* homozygous deletion (Additional file 2: Fig. S5C).

## Discussion

In this study, we investigated the survival impact of *TERT* promoter mutations in a large cohort of 560 IDH-mutated glioma cases with detailed patient data. We confirmed that majority of the *TERT* promoter mutations coincided with 1p/19q codeletion in IDH-mutated gliomas. However, there were notable exceptions, that is, 24 IDH-mutated tumors had *TERT* promoter mutations but not 1p/19q codeletion, whereas six tumors had 1p/19q codeletion without *TERT* promoter mutations. Multivariable analysis incorporating clinical background revealed that the prognostic impact of *TERT* promoter mutations was independent from that of 1p/19q codeletion (Table 2). In the subgroup analyses of grade II-III cases, the *TERT*-mutated-1p/19q intact group showed a favorable prognosis comparable to that of the *TERT*-mutated-1p/19q codeleted group, while the survival curve of the *TERT*-wildtype-1p/19q codeleted group was consistent with that of the *TERT*-wildtype-1p/19q intact group (Fig. 2a and c). These results of the subgroup analyses support the findings of the multivariable analyses.

A favorable prognostic impact of *TERT* promoter mutation in lower grade gliomas with an *IDH* mutation has been reported in several studies [6, 12, 14, 15]. However, whether *TERT* promoter mutations have an impact on patient survival independent of 1p/19q codeletion has not been fully investigated. We addressed this point by performing a multivariable analysis, first incorporating clinical factors. Our study also analyzed the prognosis of tumors with the “atypical” genotype of co-mutation in *IDH* and *TERT* without 1p/19q codeletion. The result of a large-scale retrospective study by Eckel-Passow et al. [12] indicated that this group of tumors had good prognosis comparable to that of triple-positive tumors, i.e., those with concurrent *IDH* mutation, *TERT* mutation, and 1p/19q codeletion. On the other hand, a follow-up of this study reported that *TERT* promoter mutation was a prognostic factor in 1p/19q codeleted cases, while the impact of *TERT* promoter mutation was not significant in 1p/19q intact cases [19]. However, in these studies, *TERT* mRNA expression was used as a surrogate for *TERT* mutational status in a considerable number of cases and, therefore, were not conclusive in their evaluation of the value of *TERT* promoter mutation as an independent prognostic marker in IDH-mutated gliomas [12, 19]. Another study involving over 300 IDH-mutated glioma cases also reported that survival of patients with *IDH*-*TERT* co-mutated tumors and grade II-III histology did not differ according to 1p/19q status [15]. Our results showed that *TERT* promoter mutations in IDH-mutated gliomas predict favorable prognosis regardless of 1p/19q status, highlighting the significant role of *TERT* promoter mutations as a prognostic marker. Significantly longer overall survival was seen in the *TERT*-mutated, 1p/19q intact, and IDH-mutated cases than in the *TERT*-wildtype, 1p/19q intact, and IDH-mutated cases, among patients with a high KPS score (90-100) in our study. Considering that even 1p/19q codeletion was not a prognostic indicator among patients with a low KPS (< 90), it appears that the relevance of molecular prognostic markers depends on the patient’s clinical factors. This needs to be considered in future studies investigating molecular markers.

Although the *TERT*-mutated, 1p/19q intact, and IDH-mutated cases showed comparable survival with that of the triple-positive cases, the histology of the former varied. Whether the definition of oligodendroglioma depends on the tumor’s histology or biological behavior anticipated by genotype, which is reflected in patient survival, is a matter for future debate. The current definition of oligodendroglial tumors in the CNS WHO 2016 prefers the latter [17]. On the other hand, the WHO classification is rapidly shifting from conventional morphology-based diagnosis to molecularly driven disease definition. Recognizing the significant impact of *IDH* mutation on the biology of astrocytic gliomas, cIMPACT-NOW update 5 has recently recommended a terminology “astrocytoma, IDH-mutated, grade 4” for the IDH-mutated diffuse astrocytic gliomas with histological/molecular features of glioblastoma, histological diagnosis over-ridden by molecular features [7]. A diagnosis should reflect the biology of the tumor, the natural course of disease, and/or response to therapy. The present study and other studies have reported that 1p/19q codeletion without accompanying *TERT* promoter mutations does not have prognostic benefit [19]. Of note, all cases with such genotype were histologically diagnosed as oligodendroglial tumors in our series. The combination of *TERT* promoter mutations and *IDH* mutations is a highly specific biomarker. Considering that very few single genetic alterations can sufficiently define a tumor type (even 1p/19q codeletion needs to be used in combination with *IDH* status), *TERT* promoter mutation may deserve recognition as a diagnostic marker as well.

The prognostic relevance of WHO grading in IDH-mutated gliomas is controversial, although it is associated with tumor aggressiveness in their wildtype counterparts [7, 18, 22, 26]. Our results showed that the survival of patients with IDH-mutated 1p/19q codeleted gliomas did not differ between WHO grade II and III cases (Additional file 2: Fig. S4A). The prognostic significance of WHO grading in molecularly proved oligodendrogliomas remains controversial; our result is comparable to another study [22] but in contrast with others [19]. As a nature of retrospective study, the differences in treatment variations including chemotherapy and radiation between WHO grading may have an impact on patient outcome. Future studies on oligodendroglial cases with controlled treatment background is warranted to assess this issue [19]. On the other hand, our results showed that the survival of patients with IDH-mutated astrocytomas differed among grade II, III, and IV tumors (Additional file 2: Fig. S4B); this result is comparable to those of some previous studies [25] but contrasts with others [18, 20]. Currently, the diagnosis of WHO grade II and III is essentially based on the mitotic index determined by microscopic observation of diffuse astrocytomas, and this has remained the same in the CNS WHO 2016 classification. Attempts to molecularly define the aggressive type of diffuse astrocytomas have suggested several genetic markers such as *RB1* pathway alterations (e.g., *CDKN2A/B* homozygous deletion or *CDK4* amplification), *PIK3R1* mutation, *PDGFRA* amplification, or G-CIMP low type in the methylation cluster [2, 3, 7, 11, 21, 25]. Of these, the *CDKN2A* homozygous deletion has been proposed as a strong prognostic factor in IDH-mutated astrocytomas [3, 21, 25]. In our series, high histological grade and *CDKN2A* homozygous deletion were adverse prognostic factors in IDH-mutated-1p/19q intact gliomas. This is in line with previous reports [21]. However, the frequency of this copy number change was relatively low and strongly correlated with high histological grades. IDH-mutated glioblastomas without *CDKN2A* homozygous deletion still showed poorer prognosis compared with that of lower grade astrocytomas (Additional file 2: Fig. S5C). WHO grade IV was an independent risk factor for survival in the multivariable analysis for all cases (Table 2) and the subsequent subgroup analysis for 1p/19q intact tumors (Additional file 1: Table S3L). Thus, histologically defined grade IV tumors may have a fundamentally different biology from grade II–III tumors [7]. Further exploration of molecular markers indicating aggressive IDH-mutated astrocytomas is warranted. In the meantime, histologically defined WHO grading still appears to have an impact on the delineation of biologically and clinically malignant astrocytomas with *IDH* mutation.

## Conclusions

Our results provide strong evidence that *TERT* promoter mutation confers a favorable prognosis regardless of the 1p/19q status in IDH-mutated gliomas. This observation was most evident in grade II–III gliomas as evidenced by subgroup analyses. *TERT* promoter mutations may not serve as diagnostic markers on their own as there are other types of IDH-wildtype glial neoplasms that may harbor *TERT* promoter mutations, including pleomorphic xanthoastrocytoma, ganglioglioma, anaplastic glioma with piloid features, and ependymoma [8]. However, very few molecular markers serve as standalone diagnostic markers for gliomas. Even *IDH* mutations or 1p/19q codeletion has to be used in combination to define a single entity of diffuse glioma [16, 20]. In line with this, it has been shown that *TERT* promoter mutations when combined with *IDH* mutation status serve as a very powerful prognostic predictor in diffuse gliomas. Given the current trend of using molecular and biological markers for diagnosis, it is worthwhile to consider *TERT* promoter mutation as a diagnostic as well as prognostic marker.

## Supplementary information

**Additional file 1**: Table S1 Detailed information of patients registered in the present study. Table S2 Cox regression analysis for survival in 1p/19q codeleted cases (n=284). Table S3 Cox regression analysis for survival in 1p/19q intact cases (n=273).

**Additional file 2**:** Fig. S1. OS of all cases (n=560) stratified by TERT (A) or 1p/19q (B) status.** Both *TERT* promoter mutation (A) and 1p/19q codeletion (B) were strongly associated with favorable prognosis in IDH-mutated gliomas. codel, codeleted; OS, overall survival; and 5yOS, 5-year-overall survival.** Fig. S2 Kaplan-Meier analysis for OS in IDH-mutated gliomas.** Overall survival (OS) of all cases (n=560) stratified by histological grade.** Fig. S3. Kaplan-Meier analysis for overall survival (OS) stratified by KPS score in histological grade II-III cases.** When the grade II-III cohort was subdivided by the KPS score, cases with a good performance status (with a KPS score of 90-100) showed a more favorable prognosis than those with a KPS score under 90. codel, codeleted; KPS, Karnofsky Performance Status; OS, overall survival; and 5yOS, 5-year-overall survival.** Fig. S4. Kaplan-Meier analysis for OS stratified by histological grade.** A. OS of 1p/19q codeleted cases (n=285) stratified by histological grade. B. OS of cases without 1p/19q codeletion (n=275) stratified by histological grade. codel, codeleted; OS, overall survival; and 5yOS, 5-year-overall survival.** Fig. S5. Kaplan-Meier analysis for overall survival stratified by CDKN2A status in IDH-mutated gliomas without -1p/19q codeletion (A) and those with codeletion tumors (B).** A. The prognostic impact of *CDKN2A* homozygous deletion (n=4) was not apparent in the group with 1p/19q codeletion, although the number of cases was very small. B. In 1p/19q intact tumors, cases with *CDKN2A* homozygous deletion (n=15) showed significantly shorter survival than those without this copy number alteration. C. In 1p/19q intact cases, WHO grade IV cases showed unfavorable prognosis even when analyzing cases without *CDKN2A* homozygous deletion. codel, codeleted; KPS, Karnofsky Performance Status; OS, overall survival; and 5yOS, 5-year-overall survival.

## Data Availability

The anonymized datasets analyzed in the present study are provided in supplementary information.

## Abbreviations

CI: Confidence interval
cIMPACT-NOW: Consortium to Inform Molecular and Practical Approaches to CNS Tumor Taxonomy-not official WHO
CNS WHO 2016: The revised 4th edition of the World Health Organization (WHO) classification of Tumours of the Central Nervous System
EOR: Extent of resection
KPS: Karnofsky performance status
HR: Hazard ratio
MLPA: Multiplex ligation-dependent probe amplification
OS: Overall survival
