# Supplementary material for: TERT promoter mutation confers favorable prognosis regardless of 1p/19q status in adult diffuse gliomas with IDH1/2 mutations
Source: Acta Neuropathol Commun. 2020 Nov 23;8:201. doi: 10.1186/s40478-020-01078-2 (PMC7685625; doi:10.1186/s40478-020-01078-2)
Supplement: Supplementary file 2 — Additional file 2: Fig. S1. OS of all cases (n=560) stratified by TERT (A) or 1p/19q (B) status. Both TERT promoter mutation (A) and 1p/19q codeletion (B) were strongly associated with favorable prognosis in IDH-mutated gliomas. codel, codeleted; OS, overall survival; and 5yOS, 5-year-overall survival. Fig. S2 Kaplan-Meier analysis for OS in IDH-mutated gliomas. Overall survival (OS) of all cases (n=560) stratified by histological grade. Fig. S3. Kaplan-Meier analysis for overall survival (OS) stratified by KPS score in histological grade II-III cases. When the grade II-III cohort was subdivided by the KPS score, cases with a good performance status (with a KPS score of 90-100) showed a more favorable prognosis than those with a KPS score under 90. codel, codeleted; KPS, Karnofsky Performance Status; OS, overall survival; and 5yOS, 5-year-overall survival. Fig. S4. Kaplan-Meier analysis for OS stratified by histological grade. A. OS of 1p/19q codeleted cases (n=285) stratified by histological grade. B. OS of cases without 1p/19q codeletion (n=275) stratified by histological grade. codel, codeleted; OS, overall survival; and 5yOS, 5-year-overall survival. Fig. S5. Kaplan-Meier analysis for overall survival stratified by CDKN2A status in IDH-mutated gliomas without -1p/19q codeletion (A) and those with codeletion tumors (B). A. The prognostic impact of CDKN2A homozygous deletion (n=4) was not apparent in the group with 1p/19q codeletion, although the number of cases was very small. B. In 1p/19q intact tumors, cases with CDKN2A homozygous deletion (n=15) showed significantly shorter survival than those without this copy number alteration. C. In 1p/19q intact cases, WHO grade IV cases showed unfavorable prognosis even when analyzing cases without CDKN2A homozygous deletion. codel, codeleted; KPS, Karnofsky Performance Status; OS, overall survival; and 5yOS, 5-year-overall survival. [file 40478_2020_1078_MOESM2_ESM.pptx]

## Slide 1
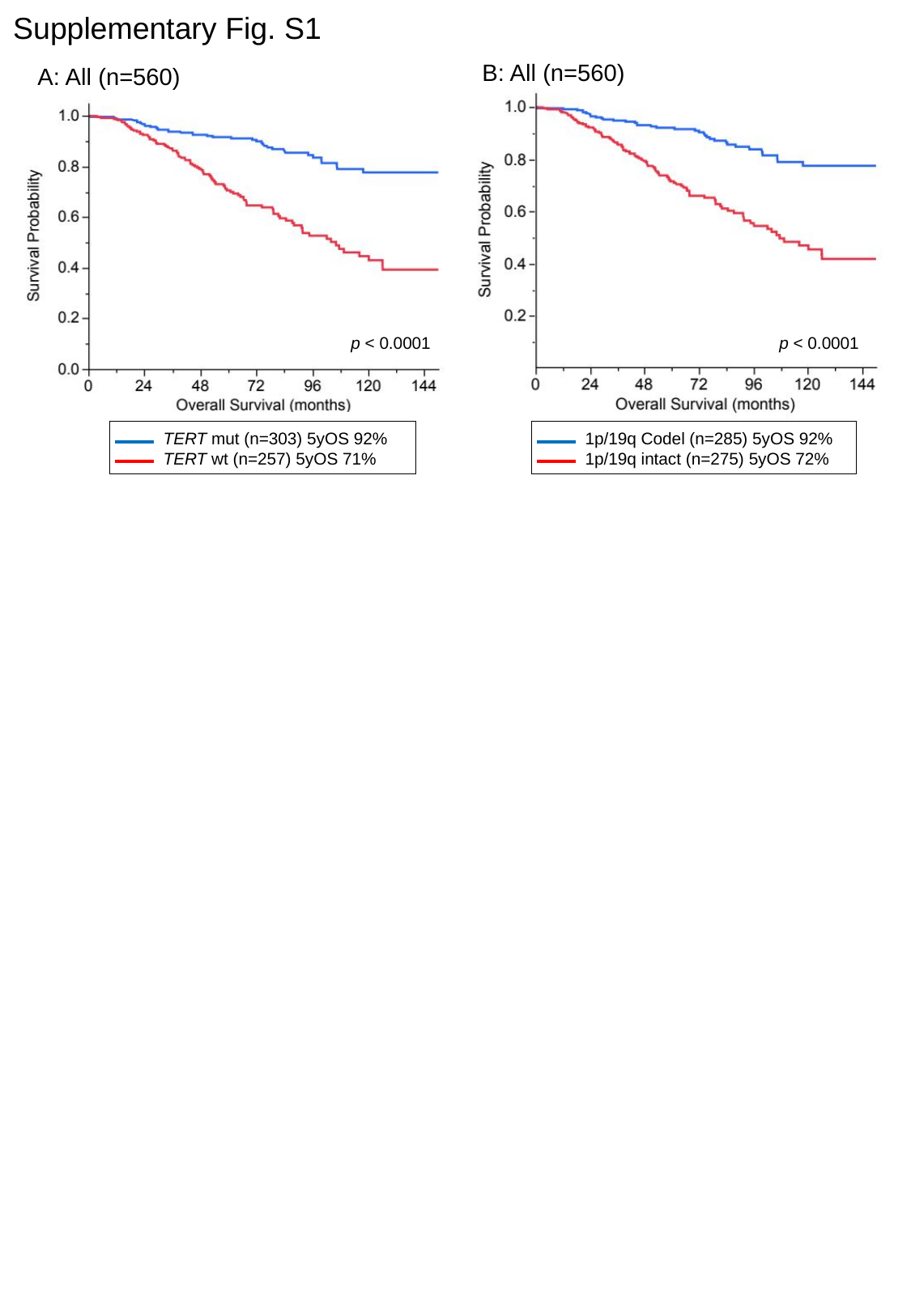

Supplementary Fig. S1
B: All (n=560)
A: All (n=560)
p < 0.0001
p < 0.0001
 TERT mut (n=303) 5yOS 92%
 TERT wt (n=257) 5yOS 71%
 1p/19q Codel (n=285) 5yOS 92%
 1p/19q intact (n=275) 5yOS 72%

## Slide 2
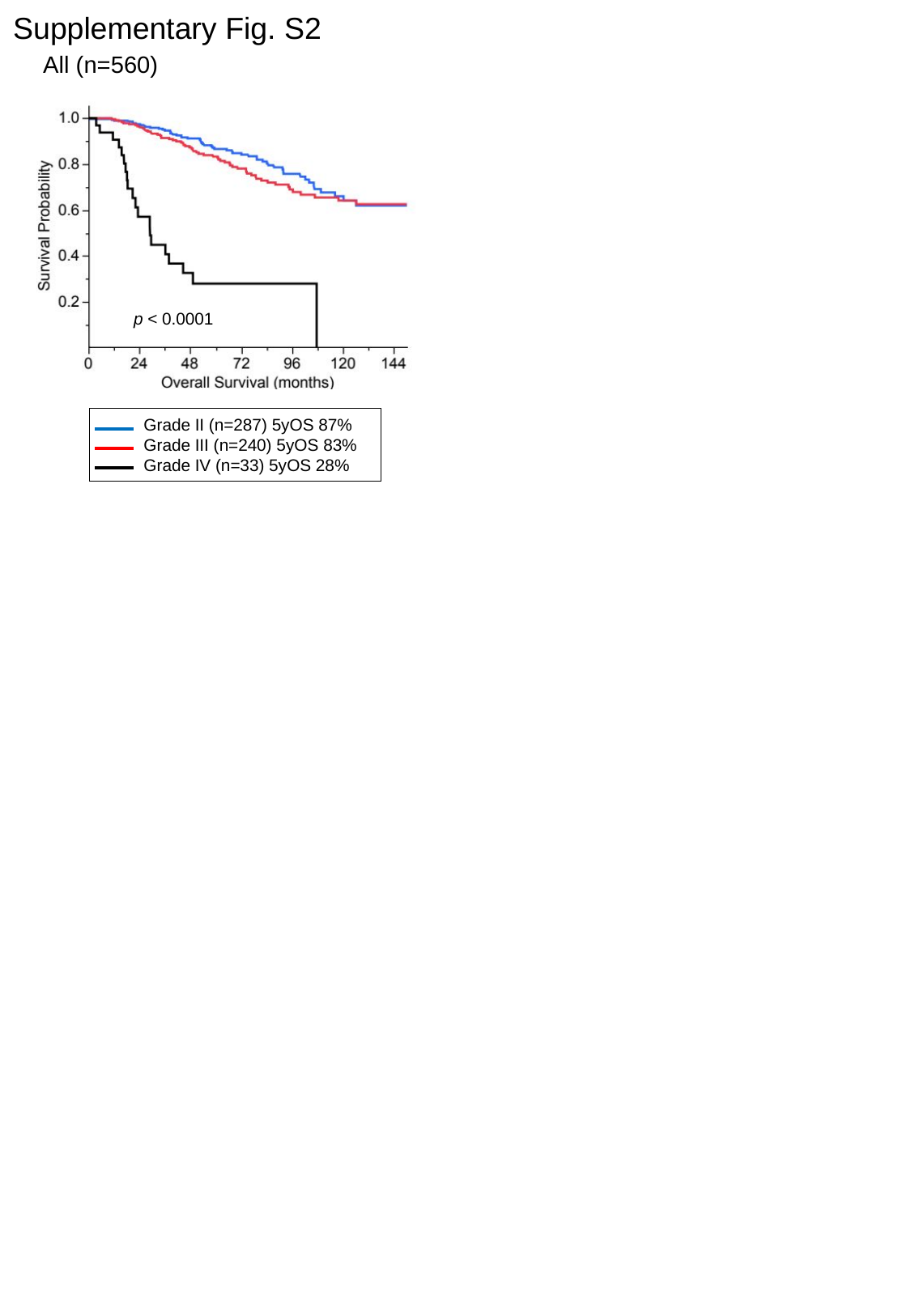

Supplementary Fig. S2
 All (n=560)
p < 0.0001
 Grade II (n=287) 5yOS 87%
 Grade III (n=240) 5yOS 83%
 Grade IV (n=33) 5yOS 28%

## Slide 3
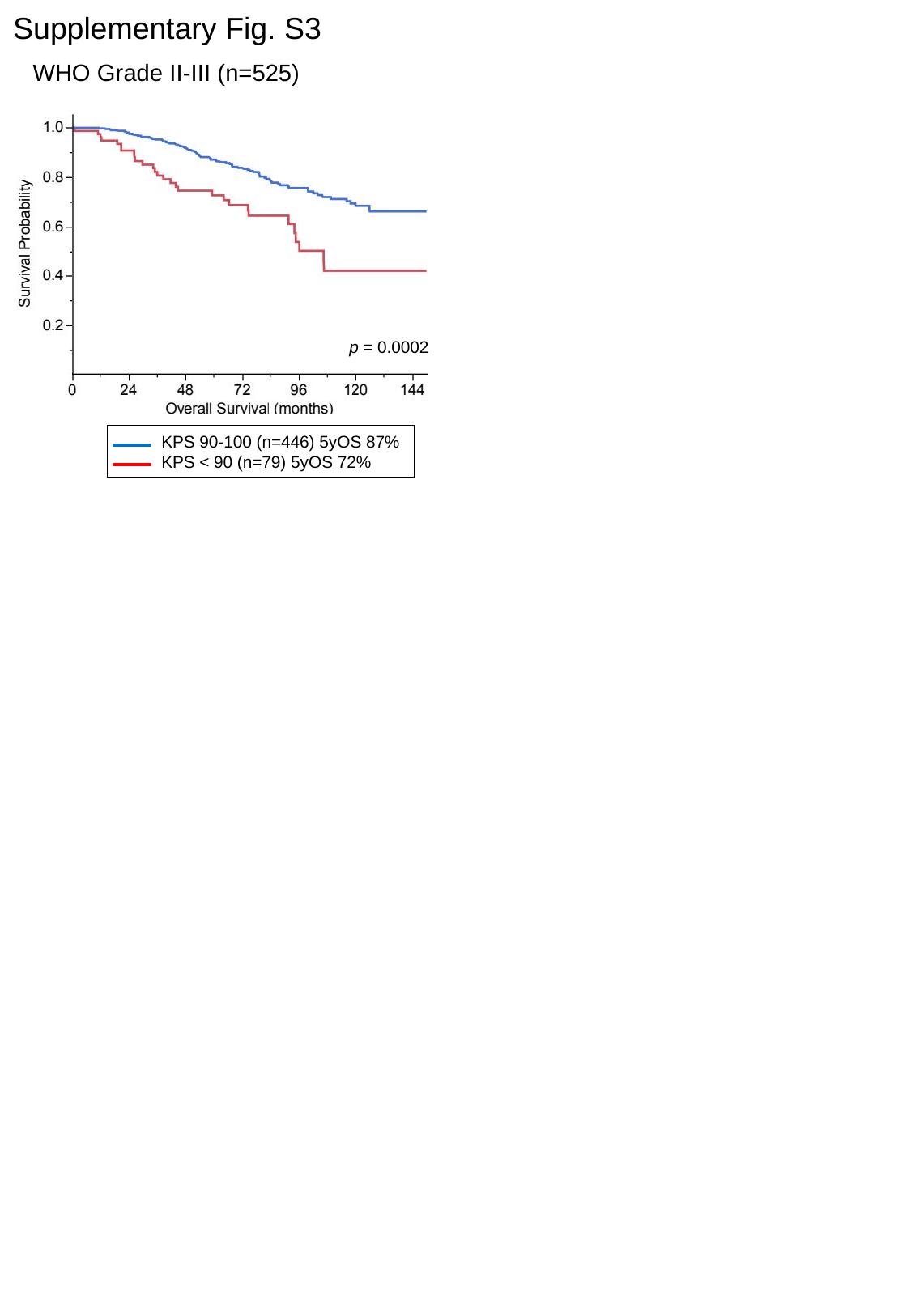

Supplementary Fig. S3
WHO Grade II-III (n=525)
p = 0.0002
 KPS 90-100 (n=446) 5yOS 87%
 KPS < 90 (n=79) 5yOS 72%

## Slide 4
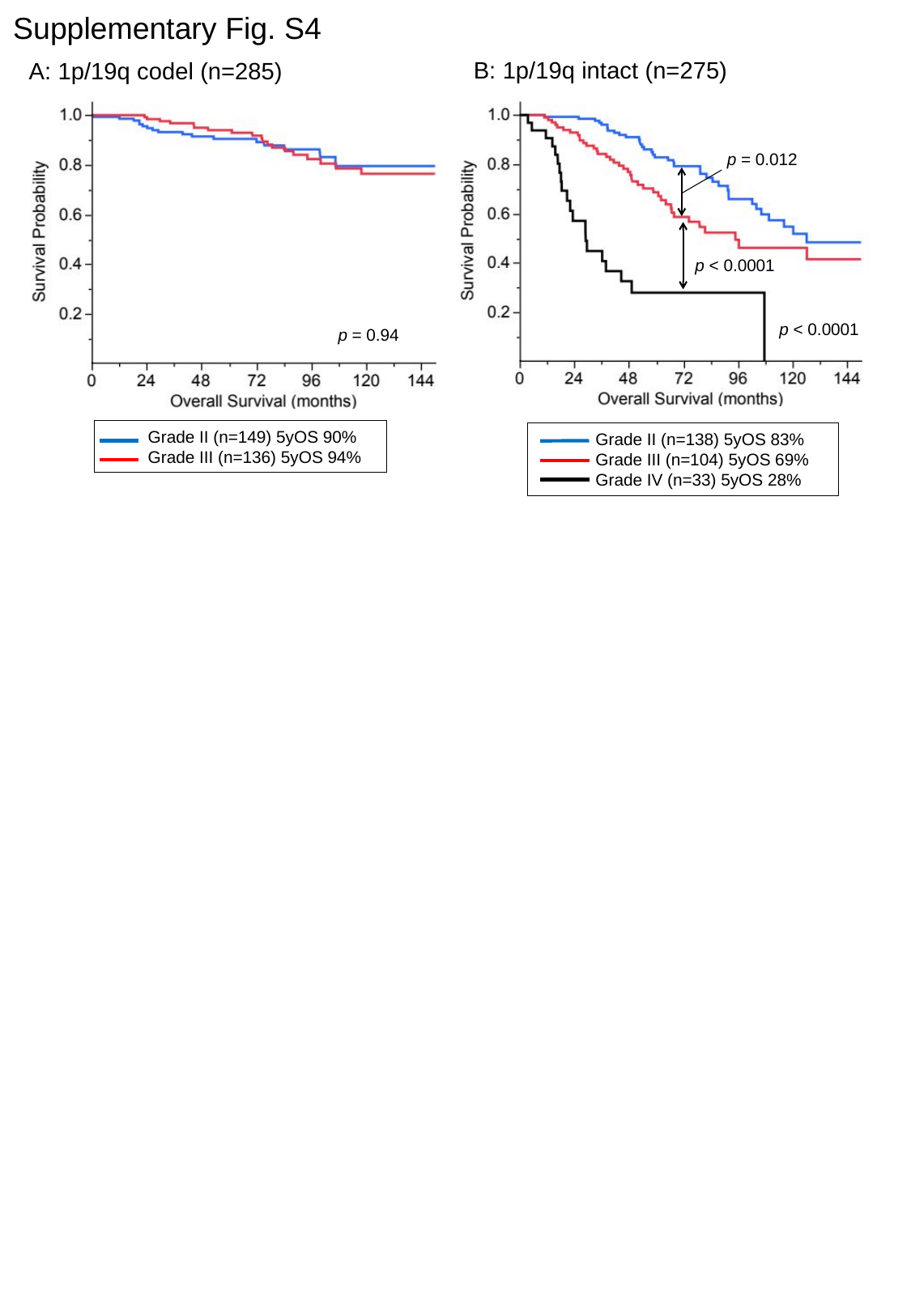

Supplementary Fig. S4
B: 1p/19q intact (n=275)
A: 1p/19q codel (n=285)
p = 0.012
p < 0.0001
p = 0.59
p < 0.0001
p = 0.94
 Grade II (n=149) 5yOS 90%
 Grade III (n=136) 5yOS 94%
 Grade II (n=138) 5yOS 83%
 Grade III (n=104) 5yOS 69%
 Grade IV (n=33) 5yOS 28%

## Slide 5
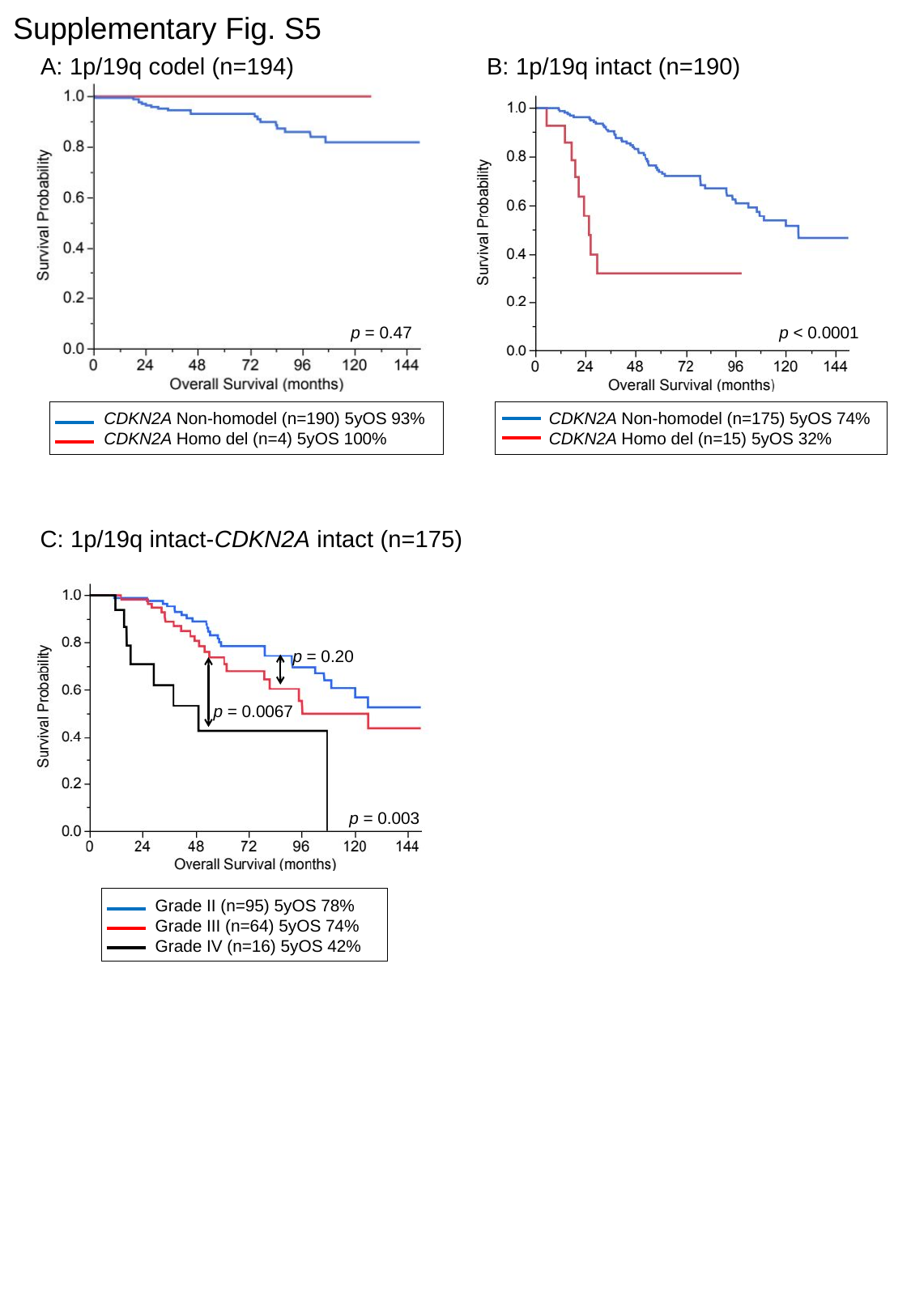

Supplementary Fig. S5
A: 1p/19q codel (n=194)
B: 1p/19q intact (n=190)
p = 0.47
p < 0.0001
 CDKN2A Non-homodel (n=190) 5yOS 93%
 CDKN2A Homo del (n=4) 5yOS 100%
 CDKN2A Non-homodel (n=175) 5yOS 74%
 CDKN2A Homo del (n=15) 5yOS 32%
C: 1p/19q intact-CDKN2A intact (n=175)
p = 0.20
p = 0.0067
p = 0.003
 Grade II (n=95) 5yOS 78%
 Grade III (n=64) 5yOS 74%
 Grade IV (n=16) 5yOS 42%
